# Vector competence of Swedish *Culex pipiens* mosquitoes for Japanese encephalitis virus

**DOI:** 10.1186/s13071-024-06269-7

**Published:** 2024-05-13

**Authors:** Janina Krambrich, Dario Akaberi, Johanna F. Lindahl, Åke Lundkvist, Jenny C. Hesson

**Affiliations:** 1 Zoonosis Science Center, Department of Medical Biochemistry and Microbiology, Uppsala University, Husargatan 3, 75237 Uppsala, Sweden; 2 International Livestock Research Institute, Hanoi, Vietnam; 3 Department of Animal Health and Antibiotic Strategies, Swedish National Veterinary Institute, Uppsala, Sweden; 4 Biologisk Myggkontroll, Nedre Dalälvens Utvecklings AB, Gysinge, Sweden

**Keywords:** Arbovirus, Vector-borne disease, Japanese encephalitis, Culicidae, Vector capacity

## Abstract

**Background:**

Japanese encephalitis virus (JEV) is an emerging mosquito-borne Orthoflavivirus that poses a significant public health risk in many temperate and tropical regions in Asia. Since the climate in some endemic countries is similar to temperate climates observed in Europe, understanding the role of specific mosquito species in the transmission of JEV is essential for predicting and effectively controlling the potential for the introduction and establishment of JEV in Europe.

**Methods:**

This study aimed to investigate the vector competence of colonized Culex pipiens biotype molestus mosquitoes for JEV. The mosquitoes were initially collected from the field in southern Sweden. The mosquitoes were offered a blood meal containing the Nakayama strain of JEV (genotype III), and infection rates, dissemination rates, and transmission rates were evaluated at 14, 21, and 28 days post-feeding.

**Results:**

The study revealed that colonized Swedish Cx. pipiens are susceptible to JEV infection, with a stable infection rate of around 10% at all timepoints. However, the virus was only detected in the legs of one mosquito at 21 days post-feeding, and no mosquito saliva contained JEV.

**Conclusions:**

Overall, this research shows that Swedish Cx. pipiens can become infected with JEV, and emphasizes the importance of further understanding of the thresholds and barriers for JEV dissemination in mosquitoes.

**Graphical Abstract:**

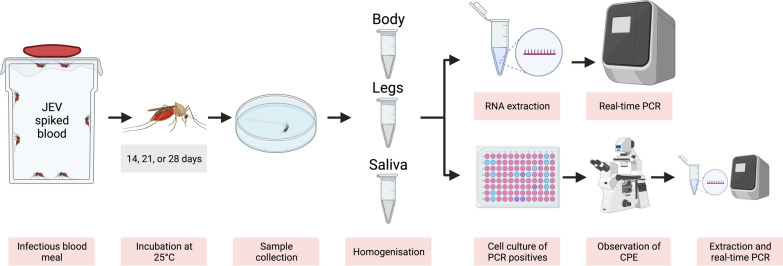

**Supplementary Information:**

The online version contains supplementary material available at 10.1186/s13071-024-06269-7.

## Background

Japanese encephalitis (JE) is an important zoonotic disease transmitted by mosquitoes. It ranks among the world's foremost causes of encephalitic illnesses, and has a particular impact on the Asia–Pacific region [1]. JE is endemic in 24 countries, ranging from Pakistan to Japan, within South and Southeast Asia, northern Australia, and Oceania, where more than three billion individuals are at risk of infection [2]. It is estimated that the annual incidence of JE is approximately 69,000 cases; however, this figure is likely an underestimate due to inadequate surveillance systems and the absence of precise diagnostic tools [3]. Notably, JE causes a substantial disease burden, resulting in an estimated annual loss of 709,000 disability-adjusted life years [4], which emphasizes the considerable public health challenge that JE presents to affected regions.

Japanese encephalitis virus (JEV) was first identified in Japan in 1935, and the virus has subsequently been reported in other Asian countries. The infection dynamics in humans are wide ranging, with seroprevalence studies suggesting that only one in 250 infections in humans is symptomatic [5, 6]. However, in 20%–30% of clinical JE cases patients die, and 30%–50% of survivors manifest severe neurological and/or psychiatric disorders [7–11].

JEV is a member of the Japanese encephalitis virus antigenic complex, and belongs to the genus* Flavivirus* within the family* Flaviviridae* [12]. It is characterized as a positive single-stranded RNA virus with a genome length of approximately 11,000 nucleotides, encoding a polyprotein that undergoes proteolytic cleavage into three structural and seven non-structural proteins [13]. Five genotypes are identified through genomic phylogeny, and they display partially overlapping distributions in Asia [14].

The transmission cycle includes pigs and wading birds, which both can serve as amplifying hosts for the virus [15, 16]. JEV relies on mosquitoes for its transmission between hosts, with species of the genus* Culex* acting as key vectors. The main JEV vector species are *Culex tritaeniorhynchus*, *Culex* *vishnui* and *Culex* *gelidus* [17–19]. On a local scale, various other species, especially within the genus *Culex*, can serve as vectors when locally abundant [20]. In Europe, *Cx. tritaeniorhynchus* has been reported in Greece [21]; however, several other *Culex* species, such as *Culex pipiens*, are widespread across the continent and serve as important vectors of other orthoflaviviruses, such as West Nile virus (WNV) and Usutu virus (USUV), as well as the alphavirus Sindbis virus (SINV) in Fennoscandia [22–28]. Recent studies have also shown that *Cx. pipiens* from France [29], China [30] and the UK can transmit JEV in a laboratory setting [31, 32].

JEV constitutes a significant contributor to viral encephalitis cases in Asia [33], and a comprehensive understanding of its potential introduction and establishment in Europe is vital for public health preparedness. JEV RNA has been detected in different samples collected in Italy, all showing maximum similarity (99%) with JEV genotype III [34]. The samples include a formalin-fixed bird tissue sample collected in 1997, six samples from birds collected in 2000, and one pool of European *Cx. pipiens* mosquitoes collected from the field in 2010 [34, 35]. Current climate models project increasing global temperatures and changes in climatic zones [36], which may render Europe more hospitable to JEV transmission [37]. Simultaneously, ongoing environmental transformations, such as urbanization and alterations in wetland ecosystems, have the potential to impact mosquito populations and their interactions with avian hosts [38]. Coupled with the heightened global mobility of both people and livestock, there is the potential for JEV to emerge in previously uncharted regions [18, 39]. Given the importance of *Cx. pipiens* as a vector for West Nile virus, USUV, and Sindbis virus in Europe, and its wide distribution in Sweden, it is important to determine the ability of local *Cx. pipiens* to transmit JEV [40–42]. In this study, we aimed to evaluate the potential of a genotype III JEV strain to infect colonized Swedish *Cx. pipiens* and assess the capability of these mosquitoes to act as vectors for JEV.

## Methods

### Mosquito strain

*Culex pipiens* (biotype molestus) mosquitoes were originally collected in Gothenburg, Sweden in 2016 [43]. The mosquitoes were reared in the mosquito laboratory of the Zoonosis Science Centre at Uppsala University under maintained conditions of 21 °C ± 1 °C and 70% ± 5% relative humidity with a 16-h light/8-h dark photoperiod. Adult mosquitoes were provided 10% sugar solution ad libitum and were given defibrinated horse blood (sourced from Håtuna lab, Uppsala, Sweden) mixed with 5% sugar on a weekly basis.

### Virus strain and cell lines

The Nakayama JEV strain (GenBank no. EF571853), a genotype III virus, was obtained from the University of Ljubljana Heritage Collection by Professor Avšič-Županc via the European Virus Archive Global (SKU reference 007V-03215). This virus was first isolated in 1935 in Japan from human cerebrospinal fluid, and has since been cultivated on Vero E6 cells. In our lab it was propagated on C6/36 cells and passage five and six were used in the described experiments.

*Aedes albopictus* C6/36 cells (Sigma-Aldrich, Darmstadt, Germany) were grown in Leibovitz's L-15 medium (L-15) (Gibco, Thermo Fisher Scientific, Waltham, MA) supplemented with 10% fetal bovine serum (FBS) (Gibco, Thermo Fisher Scientific), 10% tryptose phosphate broth (TPB) (Gibco, Thermo Fisher Scientific) and 100 units per millilitre (U/mL) each of penicillin and streptomycin (penstrep) (Gibco, Thermo Fisher Scientific) at 28 °C without CO_2_. Infected cells were incubated for 5 days in L-15 with 2% FBS, 10% TPB, and 1% penstrep at 28 °C without CO_2_. Viruses were harvested and stored at − 80 °C.

Golden hamster kidney cells clone C13 (BHK21) (Friedrich-Loeffler-Institut, Greifswald, Germany) were used for virus titration. These were grown in Dulbecco's modified Eagle medium (DMEM) (Gibco, Thermo Fisher Scientific) supplemented with 10% FBS and 100 U/mL penstrep at 37 °C with 5% CO_2_.

### Virus titration

Virus titration was performed using the 50% tissue culture infective dose per millilitre (TCID50/mL) on BHK-21 cells. Cells were seeded at a density of 1.3 × 10^4^ cells/well in 200 µL DMEM supplemented with 5% FBS and 1% penstrep in cell culture-treated 96-well plates. A tenfold serial dilution of the virus was prepared in DMEM with 2% FBS, and 100 µL of virus dilution was added per well. Virus dilutions ranging from 1:10^2^ to 1:10^11^ were tested in six wells each. The plates were then incubated at 37 °C for 3 days. On day 3, the cytopathic effect (CPE) was determined visually (Additional file 1: Fig. S1) and quantified by a tetrazole [3-(4,5-dimethylthiazol-2-yl)-2,5-diphenyltetrazolium bromide; MTT (M2128; Sigma-Aldrich)] cell viability assay, as previously described [44]. Briefly, cells were treated overnight with 10 µL of 5 mg/mL MTT solution in phosphate-buffered saline. The formazan crystals that formed were solubilized by adding 100 µL of a 10% SDS, 0.01 M HCl solution. After overnight incubation, optical densities (ODs) were read at 570 and 690 nm by using a Tecan Infinite M200 PRO plate reader (Tecan Trading, Switzerland). OD readings at different wavelengths were subtracted and the mean OD value and SD of the uninfected control was calculated. Wells with OD lower than the mean OD of the uninfected controls minus 3 SDs were considered infected (infected well OD < mean control wells OD – 3 × SD), while wells with OD equal or higher than that of the mean OD of the uninfected control minus 3 SD were considered uninfected (uninfected well OD ≥ mean control wells OD – 3 × SD). TCID50/mL was calculated using the Spearman–Kärber method, implemented as a Web tool by Lei et al. (available at https://www.virosin.org/tcid50/TCID50.html#Spearman-method) [45].

### Mosquito infection experiments

**Fig. 1:**
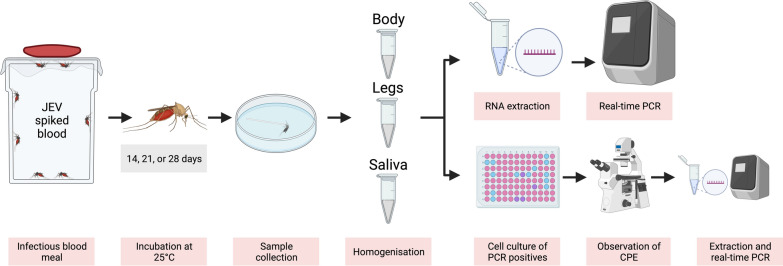
Schematic overview of the vector competence analysis workflow. Female mosquitoes were offered an infectious blood meal, a 1:4 mixture of virus suspension and horse blood. Mosquitoes were allowed to feed for 2–3 h at 25 °C in the dark. Engorged mosquitoes were incubated for 14, 21, and 28 days, representing intermediate, late, and very late stages of orthoflavivirus infection, respectively. To assess Japanese encephalitis virus (JEV) replication, body, legs and saliva were collected on days 14, 21, and 28 post-feeding. Saliva was obtained through forced salivation. Samples were transferred to microtubes containing mosquito buffer for homogenization. JEV detection was performed by using reverse transcription-quantitative polymerase chain reaction (RT-qPCR) with specific primers designed in-house. RT-qPCR-positive samples were further analysed by inoculating homogenates on C6/36 cells. Development of the cytopathic effect was monitored and supernatant collected after 3 days for subsequent RT-qPCR analysis. Workflow figure created in BioRender.com (accessed on 11 December 2023)

Mosquito infections were performed in the BSL-3 laboratory of the Zoonosis Science Centre in accordance with previous studies [23, 46–48]. Female mosquitoes aged 7–14 days were deprived of sugar solution for 24 h before being presented with an infectious blood meal. The blood meal, a 1:4 mixture of virus suspension and horse blood, had a determined viral titer of 6.81 × 10^6^ TCID50/mL for mosquitoes incubated for 14 or 21 days, and 1.47 × 10^6^ TCID50/mL for mosquitoes incubated for 28 days after a single freeze–thawing cycle. The mosquitoes were allowed to feed for 2–3 h at 25 °C in the dark, using half a cotton pad soaked with the virus-blood mixture. Fully engorged females were moved to 300-mL plastic cups and maintained at 25 °C ± 1 °C with a 16-h light/8-h dark cycle for 14, 21, and 28 days, representing intermediate, late, and very late stages of orthoflavivirus infection in this mosquito species and at the selected temperature [49]. Throughout this period, the mosquitoes had ad libitum access to 10% sugar solution. A schematic overview of the workflow is shown in Fig. 1.

To examine the replication of JEV in various parts of individual mosquitoes, the body, legs and saliva were collected on days 14, 21, and 28 post-feeding (PF) (Fig. 1). Saliva was obtained through forced salivation, as described previously [50, 51]. In brief, female mosquitoes were freeze-sedated for 5 min, and their legs were collected. The proboscis was then placed into a tube filled with 10 µL of oil for 30 min at room temperature, after which the collected saliva in oil was extracted. Subsequently, the body, legs and saliva of each mosquito were individually transferred into 1.5 mL-microtubes containing 300 µL of mosquito buffer [phosphate-buffered saline (Gibco, Thermo Fisher Scientific)] supplemented with 20% FBS, 1% penstrep, and 1% amphotericin B (Gibco, Thermo Fisher Scientific). The body and leg samples were homogenized at 25 Hz for 90 s using 5-mm stainless steel beads in a Minilys personal homogenizer bead mill (Bertin, MD).

### JEV detection in mosquito bodies, legs and saliva

Bodies of all blood-fed mosquitoes that survived the incubation period, as well as leg and saliva samples from individuals with bodies containing JEV RNA, were analysed. Viral RNA was extracted using the QIAamp Viral RNA Mini Kit (Qiagen, Hilden, Germany) according to the manufacturer’s instructions. Elution was carried out in 30 µL of water. JEV was detected in a reverse transcription-quantitative polymerase chain reaction (RT-qPCR) assay using the QuantiTect SYBR Green RT-PCR kit (Qiagen, Hilden, Germany) according to the manufacturer’s instructions. The forward primer 5′-GGCTAGCCTACAAGGTGGCG-3′ and reverse primer 5′-CTCTCGCCCATTCGGGTGAC-3′ were designed in-house. They amplify a 110-base pair-long sequence in the NS5 gene region of the JEV genome. The PCR reaction mix contained 12.5 μL of 2× QuantiTect SYBR RT-PCR Master Mix (HotStarTaq DNA Polymerase, QuantiTect SYBR RT-PCR Buffer, dNTP mix, including dUTP, ROX passive reference dye, 8 mM MgCl_2_, and SYBR Green I dye), 6.25 μL RNAse-free water, 0.5 μL forward primer, 0.5 μL reverse primer, and 0.25 μL QuantiTect RT Mix (Omniscript Reverse Transcriptase and Sensiscript Reverse Transcriptase) per reaction. A total of 20 μL Master Mix was used for each reaction well and 5 µL of extracted RNA was used as template. PCRs were run using the CFX Connect Real-Time PCR Detection System (Bio-Rad, Hercules, CA) with 30-min reverse transcription at 50 °C, 15 min initial activation at 95 °C, followed by 45 cycles of 15 s at 94 °C, 30 s at 58 °C, and 30 s at 72 °C, followed by a step-wise temperature increase from 65 to 95 °C to examine the dissociation characteristics of the amplified products. The temperature at which 50% of amplified DNA is dissociated is defined as the melting temperature, and can be observed as a peak in the graph of the negative first derivative of the melting curve. Samples were considered positive when a SYBR signal was obtained [≤ 40 threshold cycle value (Ct)], and the melting temperature was 81 °C ± 1 °C. The detection limit of the assay was between one and 10 copies/µl.

Homogenates of positive body and leg samples were inoculated on monolayers of C6/36 cells in 96-well plates to examine them for replicating virus (Fig. 1). Homogenate (100 µL) and L-15 medium (100 µL) containing 2% FBS, 10% TPB, 1% penstrep, and 1% amphotericin B were added simultaneously to the cells. Plates were incubated for 3 days at 28 °C. At day 3, cells were inspected for CPE and supernatant was collected. RNA was extracted and analysed by RT-qPCR as described above. Ct values before and after cell inoculation were compared to assess for virus replication.

### Data analysis

The number of JEV-positive bodies was divided by the total number of surviving blood-fed females at each timepoint to determine the infection rate (IR). Dissemination rate (DR), indicating the viral spread through the mosquito body, was calculated as the number of JEV-positive legs divided by the number of JEV-positive bodies. The transmission rate (TR), was calculated as the number of JEV-positive saliva samples divided by the number of JEV-positive bodies.

To identify statistically significant differences (*p* < 0.0001, *p* < 0.001, *p* < 0.01, *p* < 0.05) in infection, dissemination, and transmission across different timepoints, chi-square and simple logistic regression tests were performed using GraphPad Prism (version 10.0.0 for MacOS, GraphPad Software, Boston, MA; www.graphpad.com.)

## Results

### Feeding and survival rates of mosquitoes under laboratory conditions

Feeding rates ranged between 73 and 79%, and survival rates dropped from 90% at 14 days PF to 49% at 28 days PF (Table 1). The number of mosquitoes sampled at each timepoint ranged from 21 to 40 individuals (Table 1). All individuals for a given collection point were fed at the same time. The titers of the blood meals were equally diluted at two timepoints, once for the 14- and 21-day and once for the 28-day experiments, but subsequent titrations gave a slightly lower virus titer for the 28-day incubation (Table 1).

**Table 1:** Blood meal titres (50% tissue culture infective dose per millilitre;* TCID50/mL*) and mosquito feeding/survival rates for each timepoint

Days post-feeding	Blood meal titre	Total no. mosquitoes	Feeding rate	Survival rate
			No. fed females (percentage of total)	No. at collection (percentage of fed)
14	6.81 × 10^6^ TCID50/mL	40	29 (73%)	26 (90%)
21	6.81 × 10^6^ TCID50/mL	58	46 (79%)	40 (87%)
28	1.47 × 10^6^ TCID50/mL	55	43 (78%)	21 (49%)

### Oral susceptibility, dissemination and transmission rates of JEV

**Fig. 2:**
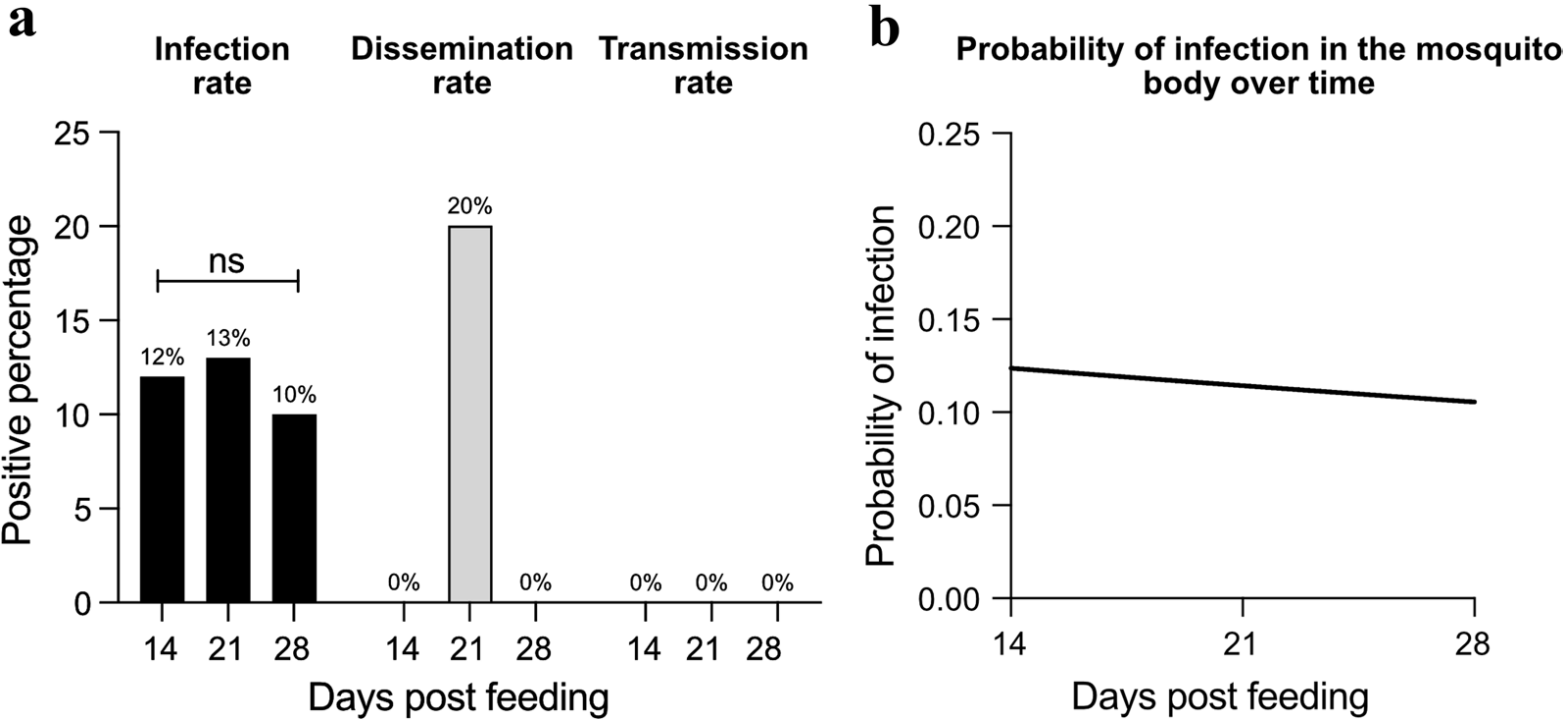
JEV-positive body, leg and saliva samples in* Culex pipiens* females following the ingestion of JEV and subsequent incubation for 14, 21 and 28 days.** a** Percentages of infected mosquitoes compared to non-infected ones after ingesting a JEV-spiked blood meal (infection rate), percentages of infected mosquitoes with JEV-positive legs (dissemination rate), and percentages of infected mosquitoes with positive saliva (transmission rate). Statistical comparison was performed using the chi-square test (*ns* not significant).** b** Simple logistic regression of the probability of mosquito body infection over time (|*Z*|= 0.20, *p *= 0.85, slope = no significant deviation from 0)

The IR, DR, and TR were calculated for *Cx. pipiens* females at 14, 21, and 28 days PF. JEV was detected in the bodies at all timepoints, in one leg sample at 21 days and in none of the saliva samples (Table 2; Additional file 2: Table S1). There was no significant difference in IR over the three timepoints [*p* = 0.942 (*χ*^2^ = 0.12, *df* = 2)] (Fig. 2a). The DR, i.e. the number of mosquitoes where JEV was also detected in the legs, was 0% at both 14 and 28 days PF, and 20% at 21 days PF (Table 2; Fig. 2a). JEV was not detected in the saliva at any timepoint in this experiment, and the TR was therefore 0% (Table 2; Fig. 2a).

**Table 2:** Infection, dissemination and transmission rates of Japanese encephalitis virus in* Culex pipiens* mosquitoes at three timepoints

Days post-feeding	No. tested	Infection rate	Dissemination rate	Transmission rate
14	26	12% (3)	0% (0)	0% (0)
21	40	13% (5)	20% (1)	0% (0)
28	21	10% (2)	0% (0)	0% (0)

The number of individuals at each timepoint is indicated in parentheses

Logistic regression was used to analyse the relationship between the incubation time and whether mosquitoes were infected or not (Fig. 2b). It showed that the probability of infection of the mosquito body was stable over time with no statistical support for a non-zero slope (*p *= 0.85). The probabilities that legs were infected could not be calculated based on only one positive sample.

To analyse if samples in which viral RNA was detected by RT-qPCR contained replicating virus, mosquito homogenate from RNA-positive samples was inoculated into C6/36 cells and the supernatant was collected and analysed for viral RNA after 3 days. CPE and a > 3 Ct value decrease was observed in one body sample and the corresponding leg sample at 21 days PF, indicating viable virus (Additional file 2: Table S1). CPE and a < 3 Ct value decrease was observed in one body sample at 14 days PF and four body samples at 21 days PF, potentially indicating viable virus (Additional file 2: Table S1). One body sample at 14 days PF showed CPE but no decrease in Ct value, and another body sample showed neither CPE nor a decrease in Ct value. The samples at 28 days PF showed CPE but no decrease in Ct value (Additional file 2: Table S1).

## Discussion

In this study, we aimed to determine the infection dynamics of laboratory colony-derived Swedish* Cx. pipiens* mosquitoes for genotype III JEV. Our findings indicate that the investigated* Cx. pipiens* mosquitoes are susceptible to JEV genotype III infection. However, at 25 °C, the virus only disseminated within one individual mosquito, and was not found in the saliva of any of the infected mosquitoes. Viable virus, CPE and a decrease in Ct value were detectable in one mosquito body after 14 days PF, in five bodies after 21 days PF, and in one leg sample at 21 days PF. CPE but no Ct decrease was observed in body samples at 28 days PF and in one body at 14 days PF. The observation of CPE for cells without detectable levels of viral RNA (by PCR) could indicate the possibility of cell death due to an unidentified factor beyond viral infection or, less likely, that viable virus may have been present in the sample but at an extremely low level that did not result in a reduction in the Ct value of the original sample. Another body at 14 days PF did not show CPE and no Ct value decrease was observed. The virus titers of the blood that was used differed slightly between the 14- and 21-day group and the 28-day group. The latter had a slightly decreased titer; however, the difference was so small that it is not believed to have had a significant impact on the results. These findings suggest that, under the conditions of our experiment, the laboratory colony-derived Swedish *Cx. pipiens* mosquitoes are not competent vectors for JEV genotype III.

The observed IR was consistent across all timepoints (Fig. 2a), whereas a disseminated infection was only observed in one sample, at 21 days PF. In mosquitoes, viruses face multiple physical barriers during infection, and it appears that JEV has limited ability to escape the midgut cells in the investigated *Cx. pipiens*. Experimental findings from other *Cx. pipiens* populations from the UK (the Wirral Peninsula), France (Montpellier and Nice) and China (Shanghai) showed that mosquitoes native to those regions are competent vectors at 18–28 °C and infectious blood meal titers of 8 × 10^6^ focus-forming unit/mL, 4.47 × 10^8^ TCID50/mL, and 1 × 10^6^ plaque-forming unit/mL, respectively [29–31]. Blood titers in the natural hosts of JEV, i.e. pigs and birds, experimentally infected with JEV are reported to range from 10^3^ to 10^5^ TCID50/mL, thus lower than the level used in most experimental studies on mosquito vector competence [52–57]. In the present study, we used titers that enabled comparison with previous experimental studies. A different study found that British *Cx. pipiens* populations could become infected with JEV at 20 °C, but that infection at a temperature of 25 °C was needed for the virus to be detected in the saliva of the mosquitoes [32]. Since JE cases in Taiwan have been associated with temperatures above 22 °C and relative humidity between 70 and 74% [58], there is potentially a critical temperature needed for JEV to disseminate and infect the salivary glands of *Cx. pipiens* [32]. However, another British study found that 72% of *Cx. pipiens* contained JEV RNA in saliva after 21 days at 18 °C, corresponding to a very high transmission rate [31]. In that study, JEV genotype II (strain CNS138-11) was used to infect the mosquitoes, while we used genotype III and did not detect any virus RNA in mosquito saliva after incubation at 25 °C. Another study examining JEV replication and sensitivity to temperature in avian and mosquito cells demonstrated that a GI-b isolate exhibited notably higher infectivity titers in mosquito cells between 24 and 48 h post-infection compared to the GI-a and GIII isolates [59]. Together, these results suggest that *Cx. pipiens* from diverse origins can become infected with JEV and, under certain conditions, as discussed above, also transmit the virus, but that the temperatures required for virus dissemination may vary between viral strains.

JEV is primarily endemic in Asia, particularly in Japan, China and Korea, and parts of Southeast Asia, and has recurrently spread into north-eastern continental Australia [60, 61]. These regions are characterized by a tropical, humid climate with consistently elevated temperatures, in contrast to temperate zones, where the climate is seasonal, with mild summers and winter temperatures that may drop below 0 °C for prolonged periods. The Nakayama strain was isolated from Japan, which is in the more temperate part of the dissemination range of JEV. JEV RNA has been occasionally detected in mosquitoes in Europe, but autochthonous transmission has never been observed [35]. Nevertheless, alterations in climate patterns have led to prolonged, warmer summers in the region [36, 62], leading to extended periods during the summer months with temperatures that support JEV transmission by native mosquitoes. Temperature and virus strains seem to influence the ability of *Cx. pipiens* to become infected and transmit JEV [29–31, 63]. Previous studies have demonstrated that laboratory colony-derived Swedish *Cx. pipiens* are capable vectors for USUV, a close relative of JEV in the *Orthoflavivirus* family, which is not currently established in Sweden [64]. Gaining insight into the vulnerability of naïve vector species in temperate zones to emerging tropical viruses is crucial for developing preventive disease intervention approaches. In addition, risk assessments for vector-borne diseases are needed when promoting changes such as increased wetland coverage to sustain increased biodiversity.

## Conclusions

Our study demonstrates that Swedish *Cx. pipiens* mosquitoes are susceptible to JEV genotype III infection. However, under the conditions of our experiment at 25 °C, the virus was inefficient in disseminating within the laboratory colony-derived mosquitoes, and was not detected in mosquito saliva. This study emphasizes the importance of gaining a deeper understanding of the thresholds and barriers for JEV dissemination in mosquitoes. Further exploration of the environmental conditions necessary for JEV transmission in temperate climates is crucial for public health preparedness, especially since the current shifts in climate and ecological landscapes may favour the emergence of JEV in previously unaffected regions. Studying viruses for which vaccinations are available is crucially highlighted not only by the reported shortage of the JEV vaccine according to agencies like the German Robert Koch Institute [65], but also by the fact that recommendations for vaccination hinge upon comprehensive risk assessment, which emphasizes the importance of understanding the vectors involved in the transmission of these viruses. Vaccine shortages further emphasize the necessity for multifaceted preparatory measures beyond merely making vaccines available, with knowledge of vectors as one essential part of this.

### Supplementary Information

**Additional file 1:** **Figure S1.** Cell cytopathic effect of JEV on BHK21 cells.

**Additional file 2:** **Table S1.** Summary of PCR results for all analysed samples.

## Data Availability

The data supporting the findings of the study must be available within the article.
