# Supplementary figures and images for: Vector competence of Swedish Culex pipiens mosquitoes for Japanese encephalitis virus
Source: Parasit Vectors. 2024 May 13;17:220. doi: 10.1186/s13071-024-06269-7 (PMC11092019; doi:10.1186/s13071-024-06269-7)

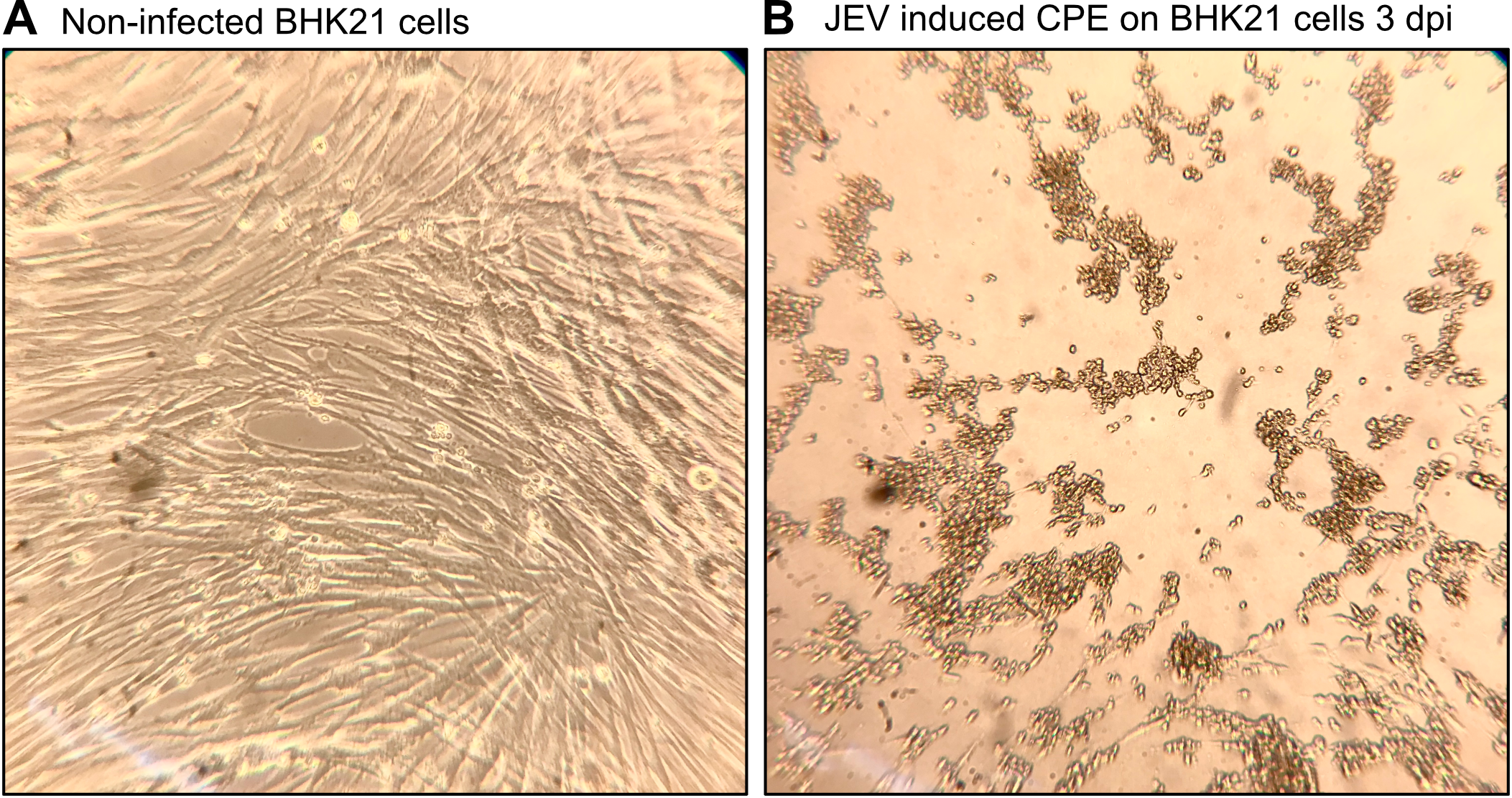

Supplement: Supplementary file 1 — Additional file 1: Figure S1. Cell cytopathic effect of JEV on BHK21 cells. [file 13071_2024_6269_MOESM1_ESM.tiff]
